# Holistic approach to visualize and quantify collagen organization at macro, micro, and nano‐scale

**DOI:** 10.1111/srt.13140

**Published:** 2022-03-14

**Authors:** Vinay Bhardwaj, Kristina Ivana Fabijanic, Aaron Cohen, Junhong Mao, Chloe Azadegan, Jean Christophe Pittet, Benedicte Le Bris

**Affiliations:** ^1^ Department of Global Personal Care and Skin Health R&D Colgate‐Palmolive Company New Jersey USA; ^2^ Orion Concept‐Technolab Tours France; ^3^ Product Development Department Filorga Laboratories Paris France

**Keywords:** anti‐aging, atomic force microscopy, collagen reorganization, dermal remodeling, extracellular matrix, reflectance confocal microscopy (Vivascope)

## Abstract

**Background:**

There is scarcity of imaging and image processing techniques for accurate discrimination and quantitation of the dermal extracellular matrix (ECM), primarily collagen. The aim of this study was to develop and demonstrate a holistic imaging and image processing approach to visualize and quantify collagen remodeling at the macro‐, micro‐ and nano‐scale using histochemical imaging, Reflectance Confocal Microscopy (RCM), and Atomic Force Microscopy (AFM), respectively.

**Material and Methods:**

For proof‐of‐concept, a commercial anti‐aging product known to induce collagen neo‐synthesis and re‐organization was tested *ex vivo* on human skin biopsies from two aged females.

**Results:**

Relative to untreated skin, collagen fibers (RCM) and fibrils (AFM) were longer and aligned after treatment. The content of collagen and elastin (histochemical imaging and ELISA) statistically improved after treatment.

**Conclusion:**

Based on our findings, we can conclude: (1) AFM, RCM, and histochemical imaging can accurately discriminate collagen from other ECM components in the skin and (2) the image processing methods can enable quantitation and hence capture small improvements in collagen remodeling after treatment (commercial cosmetic product with collagen organizer technology as proof‐of‐concept). The reported holistic imaging approach has direct clinical implications for scientists and dermatologists to make quick, real‐time, and accurate decisions in skin research and diagnostics.

## 1 INTRODUCTION

Controlled remodeling of the dermal extracellular matrix (ECM) is essential for normal development and homeostasis of the skin and other organs. ECM remodeling is the hallmark in the pathophysiology of skin aging,^1^ wound healing, and some deadly diseases including but not limited to cancers and fibrosis.^2^ There are numerous aesthetic and medical options to treat these skin conditions, however, there is scarcity of imaging methods for real‐time visualization of ECM remodeling, primarily collagen that is the most abundant structural component of the dermal ECM. Although under development, some of the novel non‐invasive real‐time clinical imaging techniques to visualize collagen and its organization are Multi‐Photon Microscopy with Second Harmonic Generation (MPM‐SHG),^3^ Reflectance Confocal Microscopy (RCM),^4^ Optical Coherence Tomography (OCT),^5^, ^6^ Line‐field Confocal Optical Coherence Tomography (LC‐OCT) based on the combination of modalities of RCM and OCT,^7^ magnetic resonance imaging (MRI),^8^ and ultrasound imaging.^9^ Of these, RCM, MPM, and LC‐OCT are CE‐certified skin imaging instruments. However, among them all, the RCM technique (Vivascope^®^ 1500 and 3000 from Lucid, Inc. USA) is the only FDA‐approved clinical dermatology diagnostic technique (510(k)# K080788), which is also covered by most insurances in the USA to diagnose skin lesions.^10^ It is a non‐invasive and cost‐effective alternative to classical biopsy and histopathology techniques to diagnose and monitor skin cancers and their treatment.^10^ Among pre‐clinical imaging methods to visualize collagen organization, atomic force microscopy (AFM),^11^ electron microscopy,^11^, ^12^ histochemical imaging using fluorescence microscopy,^13^ polarized light microscopy,^14^ confocal laser scanning microscopy (CLSM),^15^, ^16^ multimodal confocal reflectance and fluorescence microscopy,^17^ and small‐angle X‐ray scattering^18^ are well known.

One of the major caveats that restrict the use of the above‐mentioned clinical imaging techniques is that it requires tedious manual assessment because subject matter expertise and experience to accurately analyze black and white images of skin structures is a prerequisite.^19^, ^20^ Therefore, more efforts are required in the field of image processing to develop and validate algorithms and software to automate analysis,^5^ produce easy‐to‐interpret digitally stained images,^16^, ^17^ and extract quantitative information to monitor disease progression and therapy, including collagen organization in context to our research.^3^, ^6^, ^12^, ^13^, ^14^ It will enable non‐dermatologists and dermatologists to make decisions faster and with more confidence as the decisions will be made from a larger sample size of the randomized images. Some fundamental studies have been done to show use of RCM and MPM to understand age‐related changes in skin (including collagen organization) in young versus old or photo‐aged subjects.^21^, ^22^ However, there is limited effort in expanding the power of these imaging techniques to capture improvement in treatment, which requires sophisticated image processing to obtain quantitative information.^3^, ^13^, ^16^

The aim of this study was to develop and demonstrate a holistic imaging and image processing approach to visualize and quantify improvements in dermal remodeling (primarily collagen). For proof‐of‐concept, a clinically‐proven commercial anti‐aging cosmetic product with collagen organizer technology was used to assess the feasibility of using image processing to quantify minor changes in collagen before and after treatment.

## 2 MATERIALS AND METHODS

### 2.1 Skin biopsies and their treatment

Skin biopsies used in this research were the leftover material from the abdominal plastic surgery obtained from two donors after their consent (consent retained by the clinics). Donor#1 (62 year old) and donor#2 (52 year old) were both females and Fitzpatrick skin type II. Biopsies were maintained under standard culture conditions and treated daily with test product or control (untreated or placebo) for 6 days, and harvested on day 7 for imaging and ELISA. Biopsies from donor#1 was used for ELISA, RCM, and AFM, while biopsies from donor#2 were used for histochemical imaging.

### 2.2 RCM and AFM imaging

The skin biopsies from donor#1 were harvested on Day 7, and imaged directly without any sectioning and staining. AFM (Bruker, Multimode 8 AFM) and RCM (Vivascope® 1500) were used to obtain high‐resolution nano‐ and micro‐scale images of collagen in skin treated with the test product, and untreated as control. For all experiments, the AFM was equipped with a small cantilever (PPP‐FMR‐20, Nanosensors): spring constant, *k*  =  0.5–9.5 N/m, resonance frequency, *f*  =  45–115 kHz in air and was operated in the tapping mode at the room temperature.  The skin samples were mounted laterally onto a magnetic disk (1 mm ×1 mm) and placed on the stage.  The AFM images were acquired through lateral/side view of the skin biopsies to avoid sectioning. For RCM imaging, seven random images were acquired from treated and untreated biopsy from top (Stratum corneum side) and bottom (dermis side) to acquire high‐quality images of collagen. RCM images were processed and analyzed using ConfoScan® for collagen texture to report the mean fragmentation index. The fragmentation index is defined by the area of objects divided by number of objects obtained after processing raw image for collagen texture. Figure S1 shows image processing using ConfoScan® to obtain quantitative values on collagen fragmentation index (CFI).

### 2.3 Histochemical imaging

To test the effect of anti‐aging product on treating photo‐aged skin (recovery of photo‐damaged collagen and elastin), the biopsies from donor#2 were exposed to a simulated dose of UV (6 J/cm^2^ with 96% UVA). The samples and conditions tested in the experiments were: (A) negative control (untreated, no UV, and no test product), (B) positive control (skin exposed to UV, but no test product), and (C) treated (skin exposed to UV, followed by daily treatment with test product). Skin biopsies were harvested on Day 7, sectioned, stained for collagen (Picosirius staining) and elastin (immunostaining), and imaged. The images were processed and analyzed using a proprietary image analysis algorithm to obtain quantitative information on the content of collagen and elastin in papillary dermis. Briefly, the analytical process to obtain quantitative information on collagen and elastin contents include conversion of RGB images to LAB color space, filtering out background to obtain clean images on collagen and elastin, and then normalizing the collagen and elastin content in the papillary dermis to same area or number of pixels. There were six biopsies or skin samples for each condition and two sections or images from each sample, resulting in *N* = 12 images and data points for statistical testing. Figure S2 shows the schematic of image processing approach to obtain quantitative values on the collagen content.

### 2.4 ELISA

After tissue harvest on day 7, a 4 mm diameter punch was used to obtain smaller biopsies, and selected two biopsies with ∼25 mg/biopsy. The punched biopsies (50 mg total weight) were mixed in a lysis buffer containing 0.1% Triton and protease inhibitor cocktail, followed by homogenization of the tissue using an automated dual processing homogenizer with mechanical and ultrasonic features to completely lyse the tissue. The lysed tissue was centrifuged, supernatant was collected, aliquoted into two, and stored at −80°C until used. In addition to normalization with respect to weight (50 mg), the samples were also normalized to total protein content in supernatant. The supernatant was analyzed for Pro‐Collagen 1, Elastin, Alpha‐Smooth Muscle actin (A‐SMA), Tenascin‐X, and Hyaluronic acid using commercial ELISA kits. The statistics were performed on *N* = 6 data points (3 biopsies × 2 aliquots).

## 3 RESULTS

**FIGURE 1:**
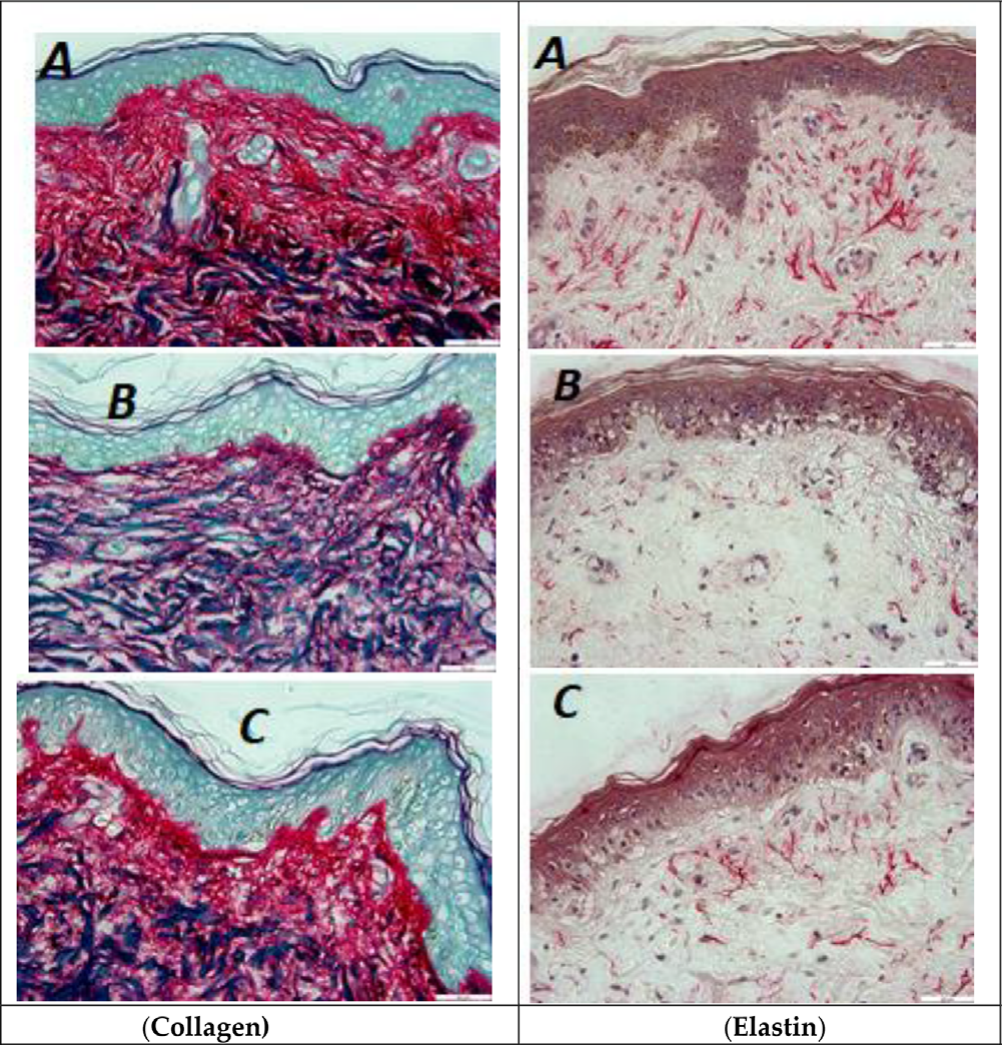
Histochemical imaging of skin sections showing collagen (purple‐red, Left row), elastin (pink‐red, right row). (A) Negative control (untreated): native skin with no treatment to UV or test product, (B) positive control: untreated with test product but treated with UV, and (C) test condition (UV followed by test product. Scale bar = 50 μm. *N* = 6 × 2 (biopsies × sections)

Histochemical imaging (Figure 1) provided macroscopic information on distribution and quantity of collagen and elastin. A clear decrease in red color of the collagen and elastin fiber bundles was observed after treatment of the skin biopsies with UV.

**FIGURE 2:**
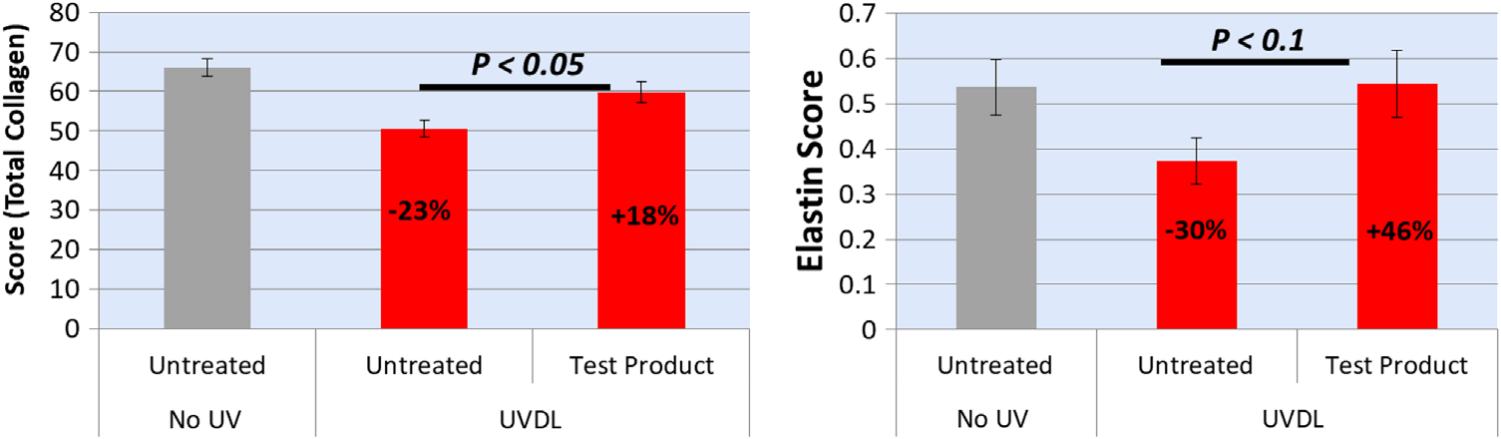
Levels of collagen (left) and elastin (right) quantified from histochemical images to compare negative control (untreated), positive control (treated with UV but not test product), and treated with UV followed by test product. *N* = 6 × 2 (biopsies × sections)

Table 1 shows the quantitative values on collagen and elastin content in three treatment conditions. These values enabled us to measure improvements in collagen and elastin content, and do statistical comparisons. The decrease in collagen (−23% vs. untreated) and elastin (−30% vs. untreated) content after UV exposure was significant (Figure 2). After treatment with the test product for 6 days the UV exposed skin biopsies were able to recover collagen (+18% vs. UV treated) and elastin (+46% vs. UV treated). Although organization of collagen is not clear in histochemical images (because the collagen is most abundant and densely packed in skin), characteristic perpendicular alignment of elastin fibers running toward epidermis is clearly observed in native (Figure 1A) and UV damaged skin after treatment with test product (Figure 1C).

**TABLE 1:** Quantitative values of collagen and elastin content in papillary dermis used for statistical comparison between treated and untreated groups (*N* = 12 images per group)

**Biomarker**	**Image#(*N*)**	**Untreated (No UV, Day 0)**	**Untreated (UV, Day 6)**	**Treated (UV+product), Day 6)**
Collagen	001	75.8	63.0	59.2
	002	80.2	38.1	59.2
	003	52.5	53.4	52.3
	004	68.3	44.2	48.9
	005	59.8	47.4	63.7
	006	64.2	43.5	40.4
	007	69.3	58.8	58.3
	008	65.6	47.1	53.1
	009	72.2	45.8	69.2
	010	59.8	58.9	72.8
	011	61.5	55.1	68.0
	012	63.2	52.2	72.7
	**Mean**	**66.0**	**50.6**	**59.8**
	S.D.	7.6	7.5	10
	SEM	2.2	2.2	2.9
Elastin	001	0.676	0.225	0.048
	002	0.298	0.259	0.338
	003	0.426	0.543	0.679
	004	0.675	0.604	0.472
	005	0.594	0.465	0.56
	006	0.222	0.694	1.06
	007	0.318	0.131	0.338
	008	0.325	0.426	0.374
	009	0.85	0.433	0.728
	010	0.714	0.186	0.705
	011	0.529	0.252	0.674
	012	0.805	0.259	0.538
	**Mean**	**0.536**	**0.373**	**0.543**
	S.D.	0.214	0.18	0.257
	SEM	0.062	0.052	0.074

The bold values in table 1 correspond to calculated mean values that are used in plotting graphs (figure 2).

**FIGURE 3:**
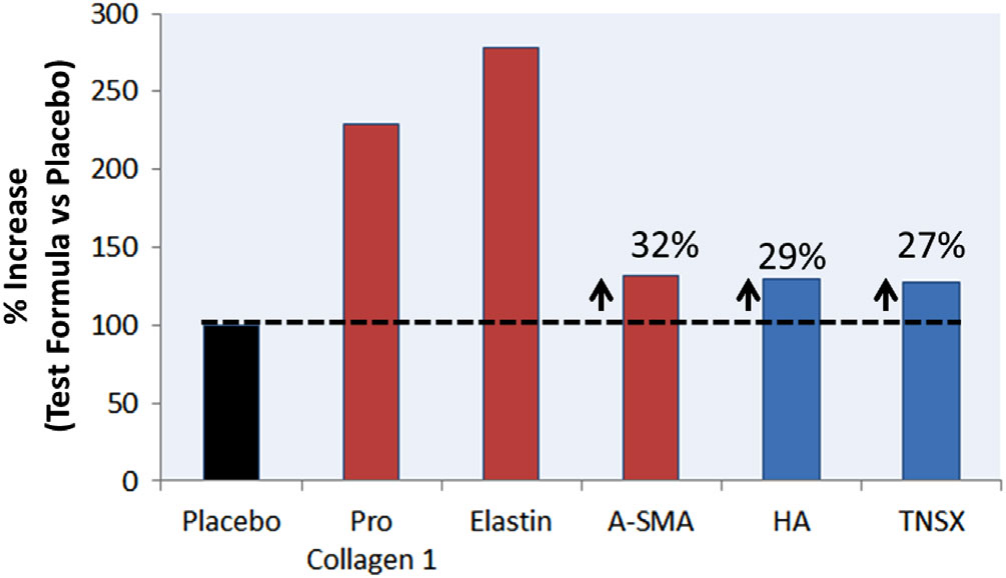
Levels of extracellular matrix (ECM) biomarkers, quantified by ELISA, reported as % increase (test product vs. placebo). Red bars highlight biomarkers with significant differences (*P* < 0.05) vs. placebo. *N* = 3 × 2 (biopsies × aliquots). A‐SMA = Alpha‐Smooth Muscle Actin, HA = Hyaluronic Acid, TNSX = Tenascin‐X

ELISA (Figure 3) compares the levels of biomarkers expressed by skin biopsies treated with the test product or placebo (that lacks a cocktail of active ingredients known for dermal remodeling). Compared with the placebo, there was 2–3 folds increase in levels of elastin and collagen, in particular type 1 pro‐collagen (test product vs. placebo). Although not significant, a noticeable increase (*P* < 0.1) in hyaluronic acid and tenascin‐X was also observed.

**FIGURE 4:**
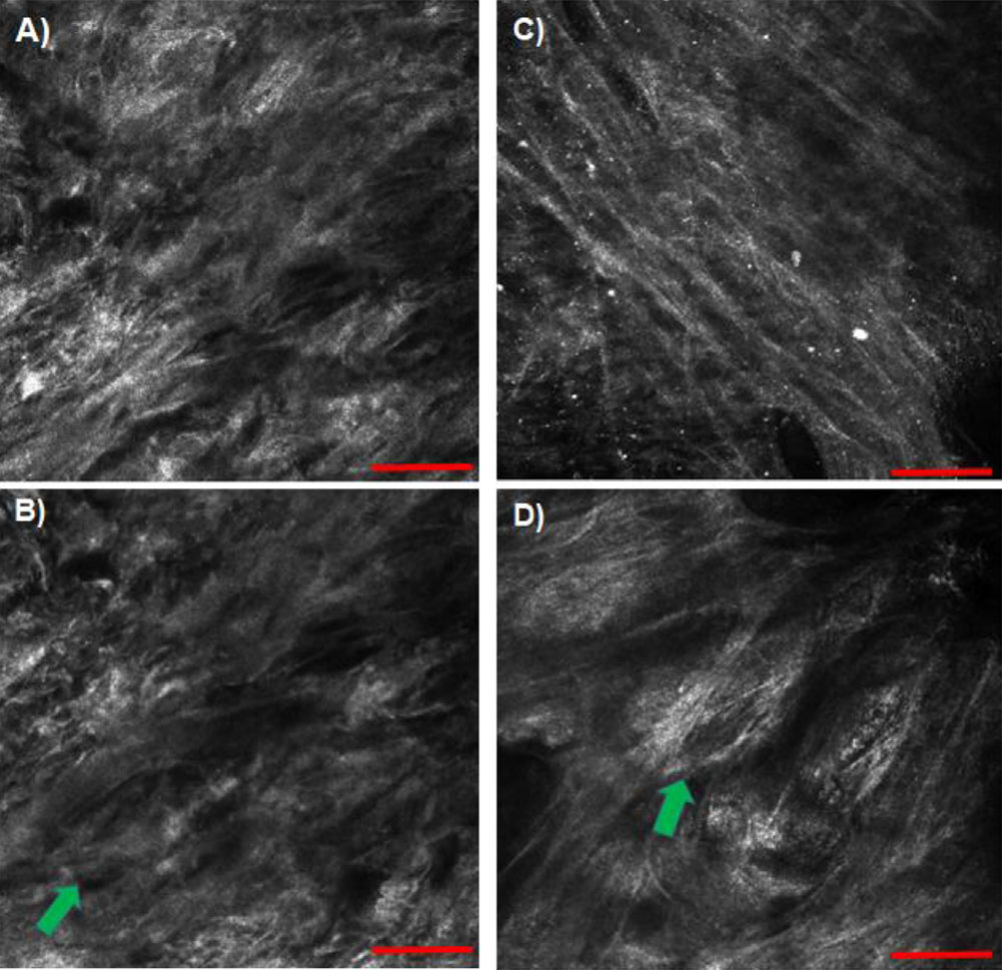
Reflectance confocal microscope (RCM) images acquired by Vivascope® 1500 showing arrangement of collagen fibers in skin, untreated (A,B) and after 6 days of treatment with test product (C,D). The arrows in the bottom images highlight fibroblasts buried inside extracellular matrix (ECM). Scale bar = 100 μm

The RCM was able to successfully reveal the organization of collagen fibers (bundle of collagen fibrils) in the skin (Figure 4). Owing to its confocal and quarter wave plate optical features, the technique was able to successfully discriminate collagen (strong endogenous contrast agent with bifringence) from other matrices without sectioning and staining the skin. The short fragmented collagen and its huddled arrangement (characteristic of damaged and poorly organized collagen in aged/photo‐aged skin) is observed in untreated skin biopsy. After treatment with the test product for 6 days, the arrangement of collagen fibers in this 62‐year‐old female skin biopsy (donor #1) looks relatively more organized than untreated, collagen fibers with length more than 100 μm running parallel to each other. Although poor contrast, we can observe the shape and size of the fibroblasts (highlighted by arrows in Figure 4), large and spread fibroblast with regular shape in the treated skin vs. untreated skin. Bright round cells in Figure 4C are of particular interest. They could be mast cells or inflammatory cells. It is not clear whether these inflammatory cells are representative of normal skin health condition or they were expressed in response to significant force applied on the laser head to try to achieve better contact between laser head and skin for high‐quality images of the collagen fibers.

Table 2 shows the mean fragmentation index determined by ConfoScan® analysis of seven random images of treated versus untreated skin. Collagen mean fragmentation index of treated versus untreated group was 0.032 and 0.064, respectively. Decrease in fragmentation index indicates improvement in collagen organization.

**TABLE 2:** Collagen mean fragmentation index calculated using ConfoScan^(R)^, to compare quality of collagen in treated vs. untreated skin biopsies. *N* = 7 random images for each group

**Treatment**	**Image/site# (N)**	**Mean area**	**Mean object number**	**Mean fragmentation index**
No	001	60.683	344	0.051
	002	62.329	353	0.050
	003	70.251	327	0.066
	004	69.608	337	0.061
	005	73.617	329	0.068
	006	78.146	308	0.082
	007	73.760	325	0.070
	Average	69.771	331.857	**0.064**
Yes	001	40.966	522	0.015
	002	42.044	505	0.016
	003	46.457	492	0.019
	004	50.048	357	0.039
	005	41.021	383	0.028
	006	50.746	295	0.058
	007	50.003	315	0.050
	Average	45.898	409.857	**0.032**

The bold values in table 2 correspond to calculated mean values that reflect fragmentation index (collagen organization before and after treatment).

**FIGURE 5:**
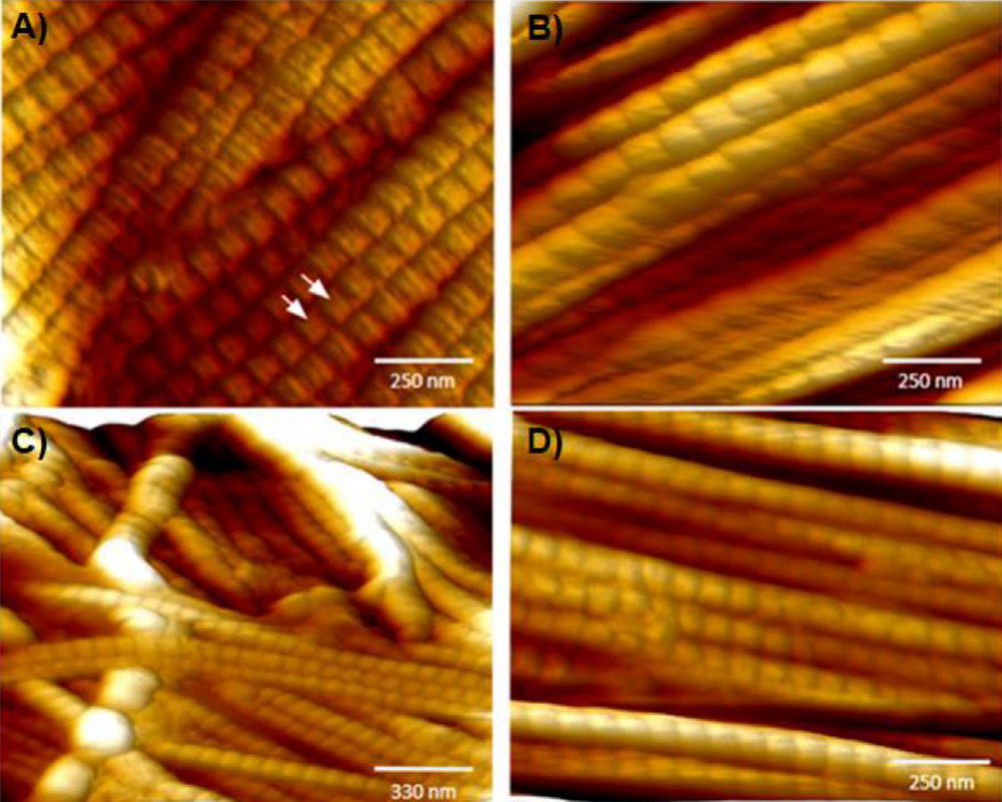
Atomic force microscopy (AFM) images showing high‐resolution images of collagen in skin, untreated (A,C) and after 6 days of treatment with test product (B,D). The characteristic traverse pattern of collagen fibrils with d‐period gap/overlap spacing of ∼68 nm is shown by arrows

To take a deeper look into collagen arrangement, AFM images were acquired to visualize collagen arrangement at nanoscale (Figure 5). We can see individual collagen fibrils (that bundle to form a collagen fiber) of nanometer thickness. Further, we can also see the characteristic traverse banding pattern of collagen fibrils (*D* ∼ 68 nm), which is in alignment with the literature,^11^ and validates that AFM was able to discriminate collagen from other ECM fibers. In consensus with RCM, relatively parallel organization of collagen fibrils was also observed under AFM for treated versus untreated skin.

## 4 DISCUSSION

In this research, we explored the potential of utilizing three imaging techniques to visualize changes in collagen in human skin biopsies treated with a commercial anti‐aging product containing some benchmark synthetic peptides known to induce collagen remodeling. The histochemical imaging and RCM imaging techniques were coupled with image processing to obtain semi‐quantitative information on collagen content and fragmentation as an index to score improvement in collagen after treatment with the anti‐aging product.

Interestingly, our study showed a very strong correlation between histochemical imaging and ELISA to report significant increase in collagen and elastin levels after treatment with the test product. Type 1 pro‐collagen is the marker of newly synthesized collagen, and its over‐expression by fibroblasts in response to Matrixyl^®^ (Sederma/Croda) is a well characterized mechanism for the anti‐aging effect.^23^, ^24^ There was also a significant increase in expression of A‐SMA, a marker unique to myofibroblasts, which are specially differentiated fibroblast cells. The role of A‐SMA in fibroblast‐mediated ECM contraction and remodeling is well understood,^25^ and a direct correlation between A‐SMA expression and fibroblasts contraction activity is reported.^26^ An increase in hyaluronic acid, though non‐significant, could be primarily attributed to the presence of hyaluronic acid as a moisturizing ingredient in the anti‐aging product. The TNSX is a novel ECM protein that is localized between or at the surface of collagen fibrils in skin dermis^27^ and TNSX is reported to induce dose‐dependent collagen fibrillogenesis,^28^, ^29^ though there is controversy whether TNSX binds specifically to pro‐collagen type 1 or other collagen and ECM biomolecules as well.^28^, ^29^ A dose‐dependent increase in TNSX in response to SKINectura™ (Lucas Meyer Cosmetics), an active ingredient in the anti‐aging test product, is reported (International patent application# PCT/IB2017/056370). Therefore, it can be hypothesized that Matrixyl^®^ (Sederma/Croda) and SKINectura™ (Lucas Meyer Cosmetics) in the test product work together to facilitate synthesis and alignment of freshly synthesized collagen, pro‐collagen type 1. A significant decrease in collagen and elastin after UV‐treatment (photo‐damaged skin model), followed by their repair after treatment with anti‐aging product (levels back to native skin not exposed to UV) indicates the capability of the histochemical imaging (polarized microscopy) and image processing technique to measure small changes in the content of ECM components (Figures 1 and 2). Diligent efforts were made to try image collagen and elastin organization (after immunofluorescence labeling) using the newly purchased Thunder Leica fluorescence imaging system. However, the resolution was not high enough and hence nothing could be reliably concluded on organization. The fluorescence‐based CLSM imaging offers higher resolution than conventional widefield fluorescence imaging to allow clear visualization of collagen and elastin organization. The conventional widefield fluorescence imaging is limited by the dominance of secondary fluorescence and the thickness of the samples, which are not a concern for CLSM.

We were able to visualize collagen organization using RCM (Figure 4). RCM is a better choice than CLSM because (1) RCM does not use any label or staining (unlike fluorescence‐CLSM that requires immunofluorescence labeling) removing any chances of uncertainty or non‐specificity due to labels, and (2) RCM is a widely used clinical dermal imaging technique for collagen. An improvement in texture of collagen fiber, long and thin fibers after treatment with anti‐aging product (vs. dense and short fragmented collagen fibers in untreated skin) clearly indicates its mode of action at the structural level. The RCM resolution was high enough to even capture fibroblast cells, the collagen synthesis factory of the skin. The collagen fibers wrapped around the fibroblasts could be the newly synthesized collagen fibers as it is thinner in diameter than surrounding collagen fibers and their bundles (Figure 4). Based on ConfoScan® analysis of randomized RCM images, we can conclude that untreated skin has a much higher index of fragmented collagen compared to treated skin. Although in RCM images we can see parallel alignment of collagen fibers after treatment with the test product, it cannot be concluded unless isotropy/anisotropy ratio is computed, which was beyond the scope of this research. However, the AFM ultrahigh resolution at the nanoscale level reveals evidence of collagen alignment at the single fibrils level. Based on positive correlation between RCM and AFM images on parallel alignment of collagen fibers (untreated vs. treated), there is a strong possibility that the anti‐aging product has the claimed collagen re‐organization property. For future research, the study should be performed to monitor the same spot (collagen fibers) over the time (longitudinal study) before and after treatment with the test product to investigate whether it is the alignment of existing collagen or the newly synthesized collagen fibers that are more aligned. However, such a longitudinal study will require integration of RCM and AFM instruments to live tissue imaging capability and do a time‐lapse imaging to capture real‐time changes in collagen remodeling.

## 5 CONCLUSIONS

Based on our research findings from this proof‐of‐concept study, we can conclude that AFM, RCM, and histochemical imaging techniques are capable of monitoring changes in collagen organization at nano, micro, and macro‐scale, respectively. AFM and RCM images show evidence of collagen alignment at nano‐ and micro‐scale after treatment with the test product. The analysis of randomized RCM and histochemical images using proprietary image processing approaches further indicates that skin after treatment with the test product has lower collagen fragmentation and higher collagen densification compared to untreated. Although some effort has been put into developing and validating image processing algorithms^3^, ^5^, ^6^, ^12^, ^13^, ^14^, ^16^, ^17^ more research is required in this direction to achieve data‐driven pre‐clinical and clinical research and diagnostics decisions. Our holistic approach of applying high‐resolution and high‐content imaging techniques in combination with powerful and robust image processing algorithms and software is a step in this direction.

Although the scope of this research was limited to investigate the collagen organization in response to an anti‐aging test product on ex vivo human skin biopsy, these imaging techniques have implications in monitoring and quantification of ECM remodeling, which is hallmark of the normal development, wound healing, as well as key marker in pathophysiology of life‐threatening conditions such as fibrosis and cancer that arise from uncontrolled ECM remodeling.^2^

## CONFLICTS OF INTEREST

The authors declare that there is no conflict of interest that could be perceived as prejudicing the impartiality of the research reported.

## Supporting information

SUPPORTING INFORMATION
