# Supplementary material for: Holistic approach to visualize and quantify collagen organization at macro, micro, and nano‐scale
Source: Skin Res Technol. 2022 Mar 14;28(3):419–26. doi: 10.1111/srt.13140 (PMC9907653; doi:10.1111/srt.13140)
Supplement: Supplementary file 1 — SUPPORTING INFORMATION [file SRT-28-419-s001.docx]

Supplementary Info


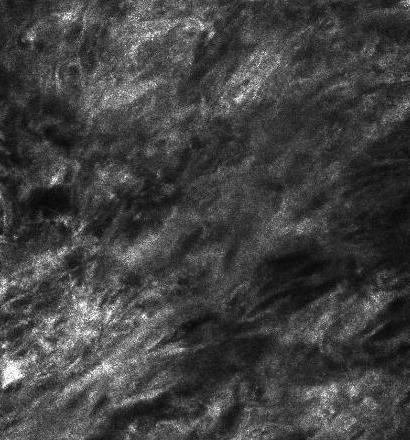

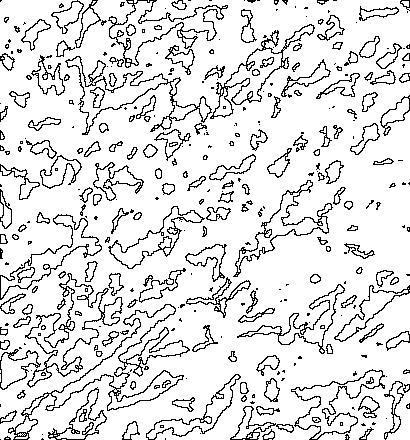


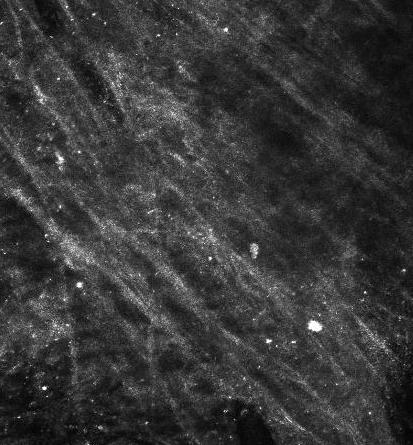

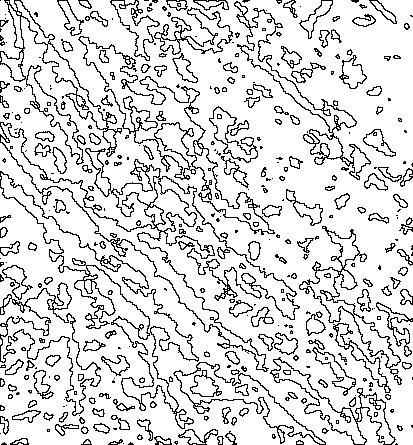


Suppl Figure 1 (S1): Illustration of vivascope image analysis by ConfoScan® for collagen texture analysis (collagen fragmentation index). Untreated (top), treated (bottom).


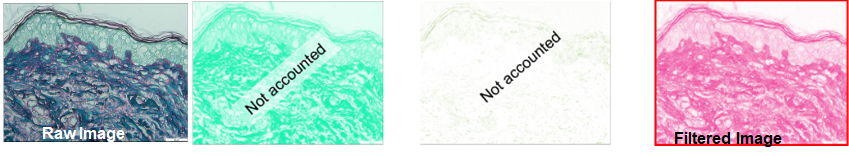


Supl Figure 2 (S2): Illustration of histochemical image processing approach for collagen analysis: Raw histochemical image is filtered to remove background signals from non-collagenous structures that are stained by Picosirius red stain. For illustration purposes, the image with high background signals was chosen.
